# Research into the reaction process and the effect of reaction conditions on the simultaneous removal of H_2_S, COS and CS_2_ at low temperature

**DOI:** 10.1039/c7ra12086a

**Published:** 2018-02-12

**Authors:** Xin Sun, Haotian Ruan, Xin Song, Lina Sun, Kai Li, Ping Ning, Chi Wang

**Affiliations:** Faculty of Environmental Science and Engineering, Kunming University of Science and Technology Kunming 650500 P. R. China likaikmust@163.com ningpingkmust@163.com +86 871 65920507 +86-871-65920507; Faculty of Chemical Engineering, Kunming University of Science and Technology Kunming 650500 P. R. China; Guangdong Provincial Key Laboratory of Environmental Pollution Control and Remediation Technology, Sun Yat-sen University P. R. China

## Abstract

In this work, tobacco stem active carbon (TSAC) catalysts loaded on to CuO and Fe_2_O_3_ were prepared by a sol–gel method and used for the simultaneous removal of hydrogen sulfide (H_2_S), carbonyl sulfide (COS) and carbon disulfide (CS_2_). The influences of the operating conditions such as reaction temperature, relative humidity (RH), O_2_ concentration, and gas hourly space velocity (GHSV) were discussed. DRIFTS results showed that the deactivation was attributed to the generation of S and sulfates. H_2_O promoted the generation of sulfate. The enhancement of the hydrolysis of COS/CS_2_ was due to the promotion of H_2_S oxidation by O_2_. A high GHSV decreased the contact time between the gases and the catalyst. Meanwhile, a high GHSV was not conducive to the adsorption of gases on the surface of the catalyst. XPS results indicated that the deactivation of the catalyst was attributable to the formation of S containing components, such as thiol/thioether, S, –SO– and sulfate. BET results indicated that the adsorptive ability of the catalyst was related to the microporous volume and surface area.

## Abstract 2

The removal processes of COS, CS_2_ and H_2_S could be divided into two parts: a catalytic hydrolysis reaction and a catalytic oxidation reaction.

## Graphical abstract

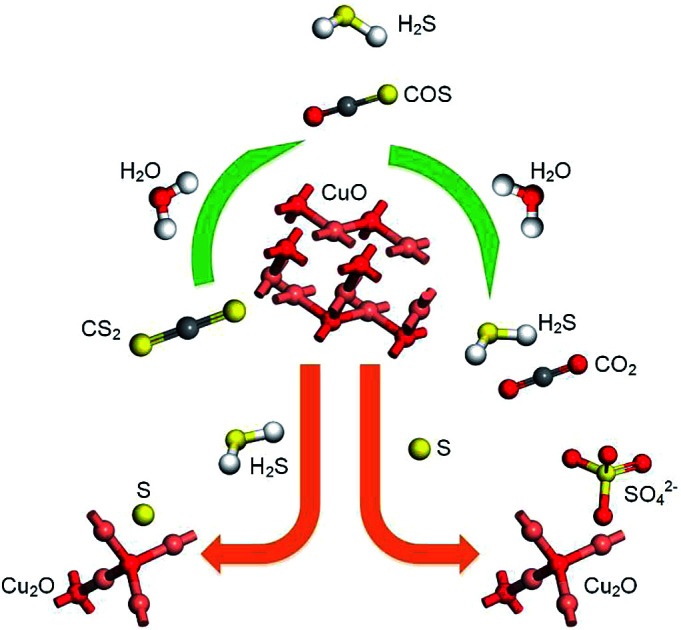

## 1. Introduction

H_2_S, COS and CS_2_ are major sulfur-containing compounds produced from industrial tail gas, such as closed carbide furnace tail gas.^1–5^ H_2_S, COS and CS_2_ not only pollute the natural environment but also cause corrosion to reactors, and poison catalysts.^6,7^ Meanwhile, there is a small amount of vapor and oxygen in the closed carbide furnace tail gas. A catalytic hydrolysis method has been widely developed to remove COS and CS_2_ from industrial tail gas.^3,8,9^ The hydrolysis product H_2_S can be removed by a catalytic oxidation method. Raw materials that have undergone desulfurization are less hazardous and corrosive, and thus can be used to produce other products. Therefore, it is of great significance to remove H_2_S, COS and CS_2_.

In previous studies, many researchers focused on the influence of catalyst characterization on the removal of H_2_S, COS and CS_2_.^1,10–14^ However, the influence of reaction conditions is also important for the removal of H_2_S, COS and CS_2_. Song *et al.* investigated the influence of reaction conditions on the hydrolysis of COS and CS_2_.^15,16^ The results showed that reaction conditions affected the reaction rate and the reaction product species. Furthermore, the reaction conditions directly affected the reaction process, such as the change of surface functional groups. In our previous studies, it could be found that –OH, –COO, and –C=O groups played important roles in the desulfurization process.^17–19^ –OH promoted the hydrolysis of COS and CS_2_, and –COO and –C=O groups promoted the oxidation of H_2_S.^20–22^ Furthermore, CO_2_ could be converted into –COO and –C=O groups during this process, which enhanced the removal of H_2_S. However, there are few studies on the simultaneous removal of H_2_S, COS and CS_2_, and the detailed changes in surface functional groups during the desulfurization process under different reaction conditions were unknown. Therefore, this study is important and valuable.

Yunnan province is the main area that produces tobacco. However, a large number of tobacco stems are discarded every year. Previous studies showed that tobacco stems could be used for the preparation of biochar, and this showed a high adsorption ability.^23,24^ Therefore, the preparation of tobacco stem biochar could solve the disposal problems of waste tobacco stems. In this work, the influence of reaction conditions (reaction temperature, relative humidity (RH), O_2_ content, and gas hourly space velocity (GHSV)) on the removal of H_2_S, COS and CS_2_ was investigated. Meanwhile, the influence of reaction conditions on the change of surface functional groups was analyzed by DRIFTS (diffuse reflectance infrared Fourier transform spectroscopy), BET (surface area and pore structure analysis) and XPS (X-ray photoelectron spectroscopy).

## 2. Materials and methods

### 2.1 Catalyst preparation

The raw material of walnut shell biochar was from Yunnan province. The main preparation parameters were firstly that the tobacco stem was washed twice with water and smashed to 4 mesh size for use in this study. Then, the walnut shell was calcined at 700 °C for 1 h under nitrogen (N_2_) conditions, and sieved to 40–60 mesh size. After that, the carbonized material and the activator (CO_2_) were mixed together, and calcined at 800 °C for 1 h.

Secondly, a colloidal solution was made with certain amounts of Cu(NO_3_)_2_·3H_2_O solution, Fe(NO_3_)_3_·6H_2_O solution and K_2_CO_3_ solution. The activated carbon catalysts were supported by the desired proportions (mass fraction of CuO was 10% and Cu/Fe = 10/1). Then, the samples were dipped into ultrasonic conditions for 30 min, dried at 100 °C in the drying oven and calcined at 400 °C at a heating rate of 5 °C min^−1^ for 4 h under nitrogen (N_2_) conditions. Lastly, the catalysts were impregnated by 5% (mass fraction) KOH, and kept under ultrasonic conditions for 10 min, then dried for 6 h at 100 °C in the drying oven to get the catalyst (Cu–Fe/TSAC) needed for the experiments. Cu–Fe/TSAC showed a high desulfurization efficiency, and the sulfur capacity was 231.28 mgS g^−1^.

### 2.2 Catalytic activity measurements

**Fig. 1:**
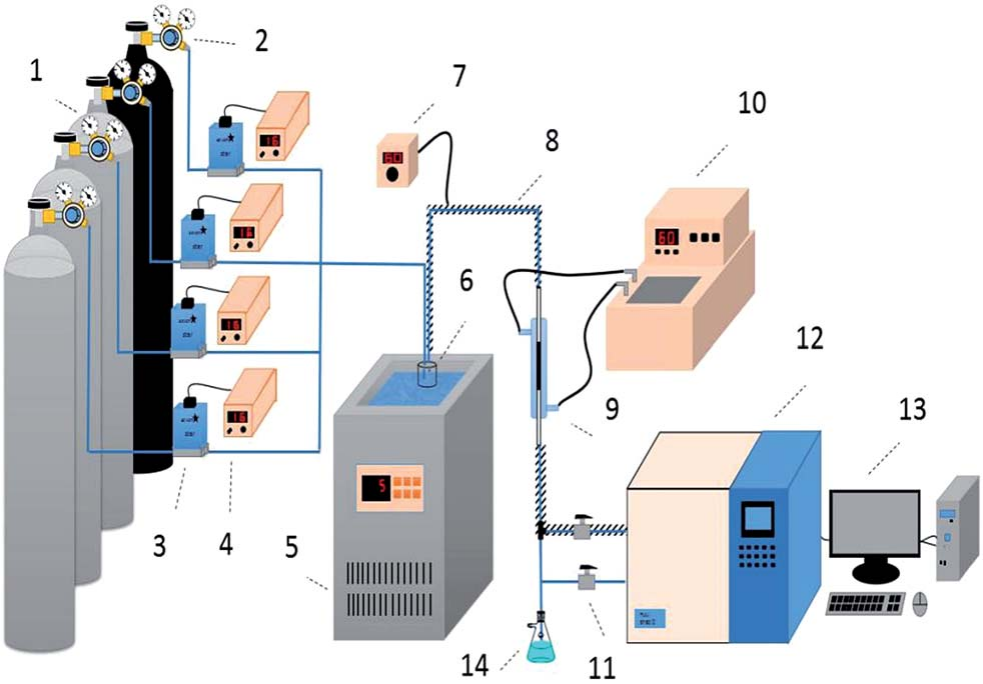
Schematic diagram of the apparatus for the simultaneous removal of H_2_S, COS, and CS_2_ ((1) compressed gases (N_2_, CS_2_, COS, and H_2_S); (2) pressure reducing valve; (3) mass flow meter; (4) flow controller; (5) low temperature thermostatic bath; (6) water saturator; (7) heating controller; (8) heating cable; (9) fixed-bed quartz reactor; (10) water bath; (11) switch; (12) FULI 9790II gas chromatography; (13) workstation; (14) absorbing bottle).

Desulfurization tests were performed in a fixed-bed quartz reactor (3 mm inside diameter, 140 mm length) under atmospheric pressure (Fig. 1). H_2_S, COS and CS_2_ from gas cylinders (1% H_2_S in N_2_; 1% COS in N_2_; 0.3% CS_2_ in N_2_) were diluted with N_2_ (99.99%) to the required concentrations (H_2_S: 500 ppm; COS: 400 ppm; CS_2_: 60 ppm). The gas hourly space velocity (GHSV) of the reaction mixture was standardized at 10 000–20 000 h^−1^. The water comes from a saturator system, and the relative humidity (RH) was 0–60%. The reaction temperature of this reactor was controlled at 50–70 °C by a water-bath with a circulating pump, with an accuracy of ±0.1 °C. FULI 9790II gas chromatography was used to analyze the total H_2_S, COS and CS_2_ concentrations of the gaseous feed and effluent from the reactor. The conversion rates of H_2_S, COS and CS_2_ are achieved according to eqn (1).1
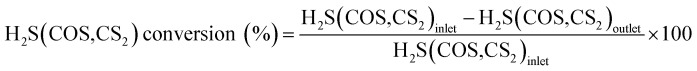


The sulfur capacity (mgS g^−1^ catalyst) is defined as the sulfur deposition per unit mass of desulfurizer agent in H_2_S, COS and CS_2_ between time points (ending at 85% conversion).

### 2.3 Characterization

DRIFTS spectra were collected using a Nicolet iS50 FTIR spectrometer equipped with a smart collector. Mass flow controllers were used to control the volume flow of different gases to the required concentrations. The heating cable controlled the temperature (70 °C) of mixed gas until it entered the reactor. A reactor heater controlled the temperature (70 °C) of the reactor in the DRIFTS experiments. In this case, it ensured that the reaction temperature of the gas phase and the solid phase are the same. IR spectra were recorded by accumulating 100 scans at a resolution of 4 cm^−1^. Nitrogen adsorption–desorption isotherms were obtained by a Quantachrome surface area analyzer instrument. Before the measurement, the samples were outgassed under vacuum at 393 K for 24 h. Specific surface areas, and mesoporous and micropore adsorption–desorption isotherms were calculated by Brunauer–Emmett–Teller (BET), Barret–Joyner–Halenda (BJH) and Horvath–Kawazoe (HK) methods, respectively. XPS (ESCALAB 250) analysis was performed using Al Kα radiation, where the energy of the Al target powered was 200 W.

## 3. Results and discussion

### 3.1 Effect of reaction temperatures on the simultaneous removal of H_2_S, COS and CS_2_

**Fig. 2:**
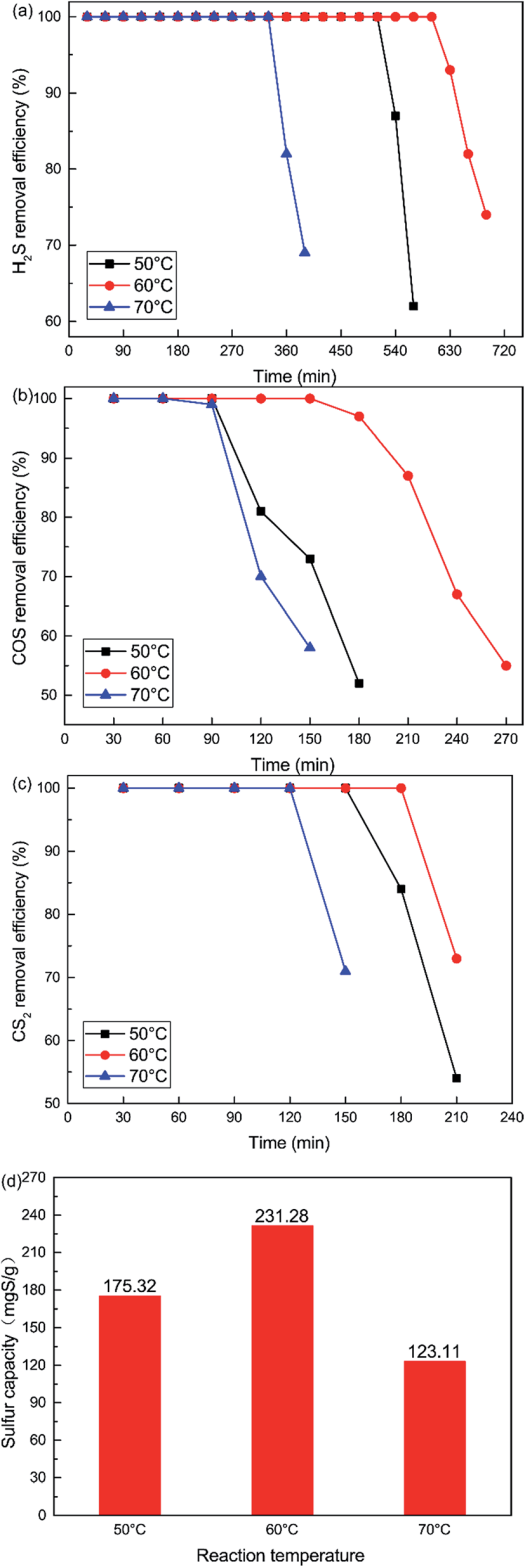
Effect of reaction temperature on the removal of (a) H_2_S, (b) COS, and (c) CS_2_, and (d) sulfur capacity (reaction conditions: 500 ppm H_2_S; 400 ppm COS; 60 ppm CS_2_; GHSV = 10 000 h^−1^; RH = 49%; O_2_ = 0%).

The influence of reaction temperatures on the catalytic performance of the Cu–Fe/TSAC catalyst is illustrated in Fig. 2. The conversion of H_2_S, COS and CS_2_ first increased and then decreased with increasing temperature, and was highest at 60 °C. The H_2_S, COS and CS_2_ conversion was 100% in the initial 600, 150 and 180 min respectively. As shown in Fig. 2(d), the sulfur capacity first increased and then decreased with increasing reaction temperatures. The highest sulfur capacity (231.28 mgS g^−1^) was achieved at 60 °C.

The reaction rates of simultaneous catalytic hydrolysis of COS and CS_2_ and catalytic oxidation of H_2_S were poor at low temperatures. Therefore, with increasing reaction temperature, the sulfur capacity was increased and the reaction rate of catalytic hydrolysis can be increased, and the hydrolysis reaction could occur more easily. However, the conversion of H_2_S to S or sulfate on Cu–Fe/TSAC involves parallel reactions. With increasing reaction temperatures, the yield rate of sulfuric acid increases faster than that of sulfur. At higher temperatures, H_2_S can be oxidized to sulfate more easily, and the higher concentration of SO_4_^2−^ poisons the hydrolysis activity.^25^ The majority of the products on the exhausted Cu–Fe/TSAC were S/SO_4_^2−^ species which accumulated on the active carbon’s surface and had a negative effect on the hydrolysis activity. Thus, the removal efficiency of H_2_S, COS and CS_2_ declined sharply at 70 °C.

**Fig. 3:**
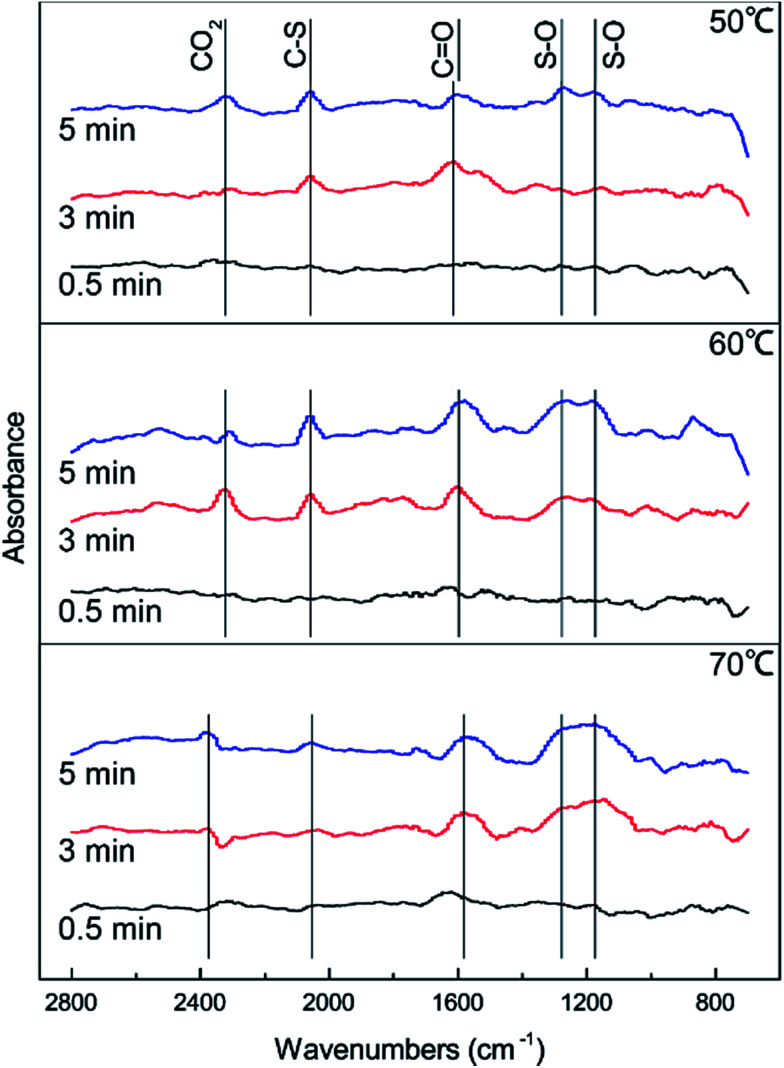
DRIFTS spectra of reactions at different temperatures (reaction conditions: 500 ppm H_2_S; 400 ppm COS; 60 ppm CS_2_; GHSV = 10 000 h^−1^; RH = 49%; O_2_ = 0%).

DRIFTS results were used to further study the catalytic reaction of Cu–Fe/TSAC at different temperatures. As shown in Fig. 3, the Cu–Fe/TSAC surface generates CO_2_ (2363 cm^−1^), C=O groups (1604 cm^−1^), C–S groups (2080 cm^−1^) and S–O groups (1140 cm^−1^ and 1307 cm^−1^) as the reaction proceeds.^1,26,27^ Furthermore, CO_2_ was produced as the reaction time progressed, which can prove that the reaction is indeed the hydrolysis of COS and CS_2_. The formation of S–O groups can prove that the H_2_S was oxidized. Compared with reactions at 60 °C and 70 °C, fewer S–O groups were generated at 50 °C. This indicated that a temperature of 50 °C was not conducive to the oxidation of H_2_S. Compared with reactions at 50 °C and 60 °C, more S–O groups and fewer C–S groups were generated at 70 °C. This indicated that a temperature of 70 °C enhanced the hydrolysis of COS/CS_2_ and the oxidation of H_2_S. However, excessive oxidation of H_2_S could generate more sulfate, which could lead to the deactivation of the catalyst. At the temperature of 60 °C, the number of C=O groups increased and the amount of CO_2_ decreased, which indicated that CO_2_ could be converted into C=O groups in the reaction. As a result, the catalyst has a good adsorptive ability for COS/CS_2_/H_2_S and a good oxidation ability for H_2_S over time. Therefore, a temperature of 60 °C is conducive to the hydrolysis of COS/CS_2_ and the oxidation of H_2_S. The result was in accordance with the activity experiment.

### 3.2 Effects of RH on the simultaneous removal of H_2_S, COS and CS_2_

**Fig. 4:**
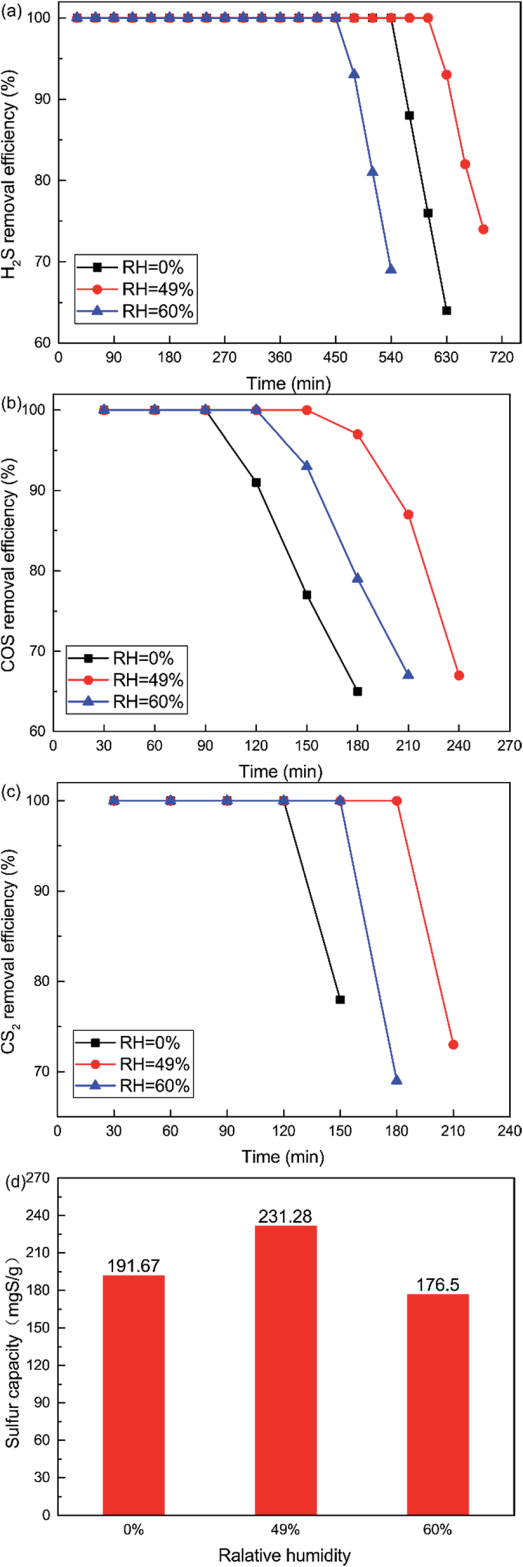
Effect of RH on the simultaneous removal of (a) H_2_S, (b) COS, and (c) CS_2_, and (d) sulfur capacity (reaction condition: 500 ppm H_2_S; 400 ppm COS; 60 ppm CS_2_; reaction temperature = 60 °C; GHSV = 10 000 h^−1^; O_2_ = 0%).

The effects of RH on H_2_S, COS and CS_2_ removal were studied by introducing feed gas through a humidifier. Influences of different RHs on the catalytic performance are plotted in Fig. 4. Removal efficiency for H_2_S, COS and CS_2_ first increased and then decreased with increasing RH. Low RH should benefit the hydrolysis and oxidation activities. When the RH was 49%, the catalyst showed the best activity, as 100% H_2_S, COS and CS_2_ conversion was maintained for about 600 min, 150 min and 180 min respectively. As shown in Fig. 4(d), the sulfur capacity first increased and then decreased with increasing RH. The sulfur capacity was highest (231.28 mgS g^−1^) when the RH was 49%. The sulfur capacity decreased to 176.50 mgS g^−1^ at the RH of 60%. The selective catalytic oxidation of H_2_S to S or HS^−^ will be easier in the presence of less vapor. The fact that excessive water could restrain catalytic activity might be due to competition between H_2_S (COS or CS_2_) and vapor for the same active sites of the catalyst.^28^ Another reason is that the pores of the catalyst’s surface will form water films when the RH reaches a certain amount. Although the formation of water films would provide more accommodating spaces for the product, excessive water films may stop H_2_S, COS and CS_2_ diffusing on the hydrolysis center and inhibit the catalytic hydrolysis reaction.^29^

**Fig. 5:**
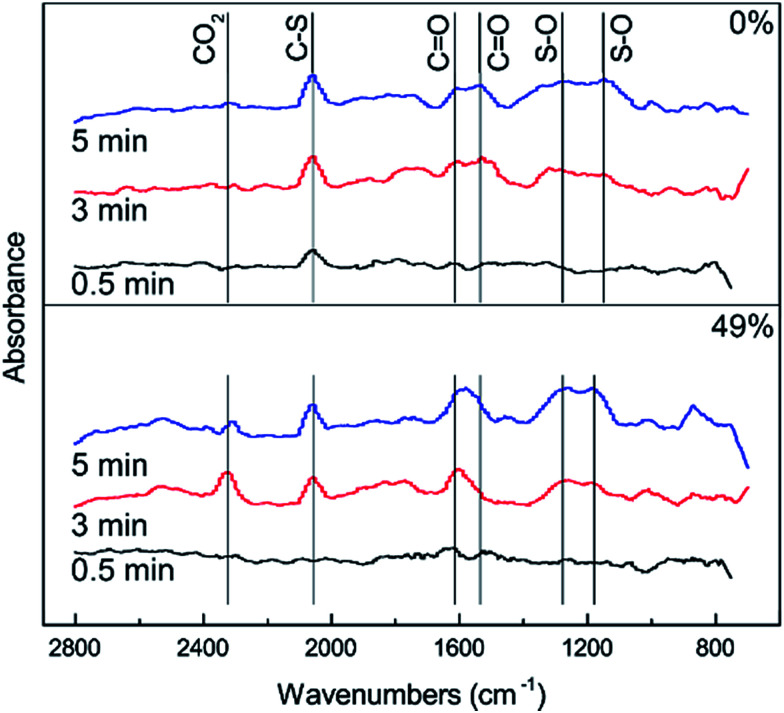
DRIFTS spectra of reactions at different RHs (reaction conditions: 500 ppm H_2_S; 400 ppm COS; 60 ppm CS_2_; reaction temperature = 60 °C; GHSV = 10 000 h^−1^; O_2_ = 0%).

In order to further investigate the effect of different RHs on the conversion of H_2_S, COS and CS_2_ over Cu–Fe/TSAC, DRIFTS measurements of the catalytic reactions over Cu–Fe/TSAC were taken. As shown in Fig. 5, there was no obvious CO_2_ peak when the reaction was performed at 0% RH. This indicated that the removal of COS/CS_2_ was an adsorption process without H_2_O.^1^ After introducing H_2_O (49% RH), a peak due to CO_2_ appeared over time. This proved that the removal of COS/CS_2_ with H_2_O was due to a hydrolysis process. Furthermore, more S–O groups were generated when the RH was 49%, which indicated that H_2_O promoted the generation of sulfate. Meanwhile, the decrease in CO_2_ and the increase in C=O groups indicated that H_2_O promoted the conversion of CO_2_. It can be deduced that the catalytic hydrolysis reaction occurs on the surface of Cu–Fe/TSAC, where the hydrolysis of COS and CS_2_ produces CO_2_ and H_2_S.

### 3.3 Effect of O_2_ content on the simultaneous removal of H_2_S, COS and CS_2_

**Fig. 6:**
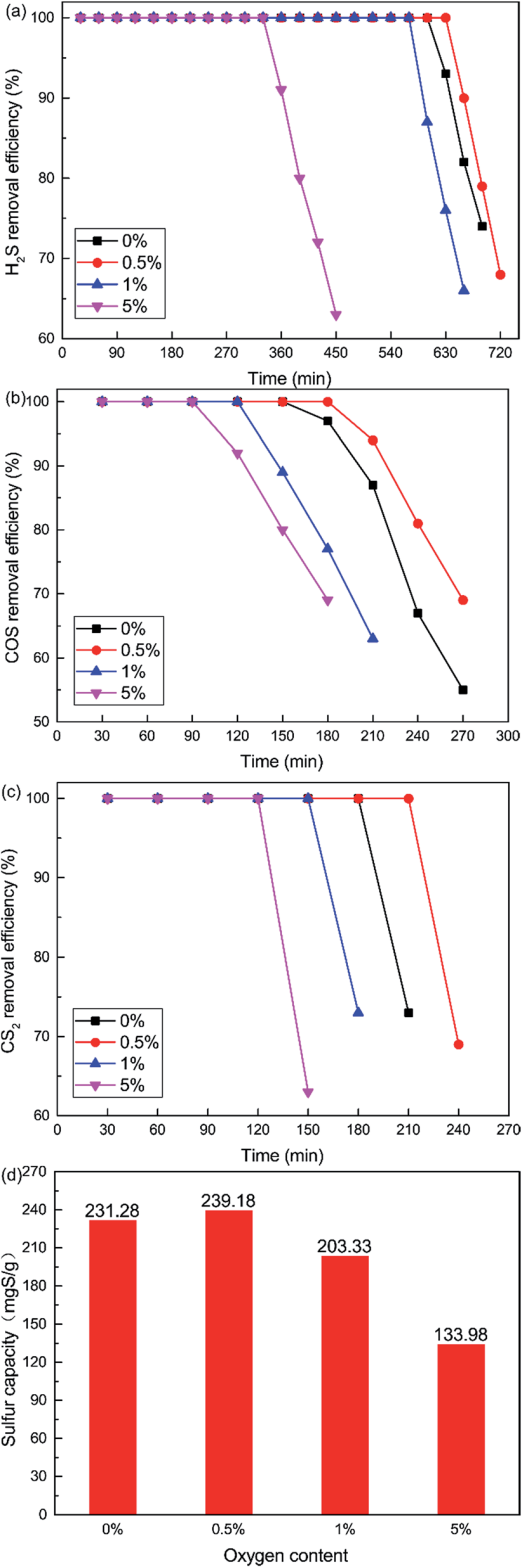
Effect of O_2_ content on the simultaneous removal of (a) H_2_S, (b) COS, and (c) CS_2_, and (d) sulfur capacity (reaction conditions: 500 ppm H_2_S; 400 ppm COS; 60 ppm CS_2_; reaction temperature = 60 °C; RH = 49%; GHSV = 10 000 h^−1^).

The curves plotted in Fig. 6 show the effect of O_2_ content on the removal efficiency of H_2_S, COS and CS_2_. It is very difficult to control the oxygen content below 0.5%, although we wanted to investigate the lower O_2_ content. So the non-oxygen conditions were investigated. What is more, the O_2_ content is extremely low, even close to zero, in closed carbide furnace tail gas which is a reductive atmosphere. The catalytic removal efficiency initially increased and then decreased with increasing O_2_ content. When the O_2_ content was 0.5%, the removal efficiency of H_2_S, COS and CS_2_ was highest. 100% H_2_S, COS and CS_2_ conversion was maintained for about 600 min, 150 min, 180 min respectively. As shown in Fig. 6(d), the sulfur capacity first increased and then decreased with the increase in O_2_ content. The sulfur capacity was 231.28 mgS g^−1^ when the O_2_ content was 0%. When the O_2_ content was 0.5%, the sulfur capacity increased to 239.18 mgS g^−1^. With a further increase in O_2_ content, the sulfur capacity decreased. The sulfur capacity was only 133.98 mgS g^−1^ when the O_2_ content was 5%.

It is clear that the addition of a little O_2_ enhanced the catalytic activity in the COS and CS_2_ hydrolysis. The reason may be that sufficient oxygen can increase the oxidation of H_2_S and promote the catalytic hydrolysis of COS and CS_2_. The oxidation rate of H_2_S speeds up with the increasing oxygen content. This will lead to the generation of more sulfate, with the inhibition effect greater than the promotion effect.

**Fig. 7:**
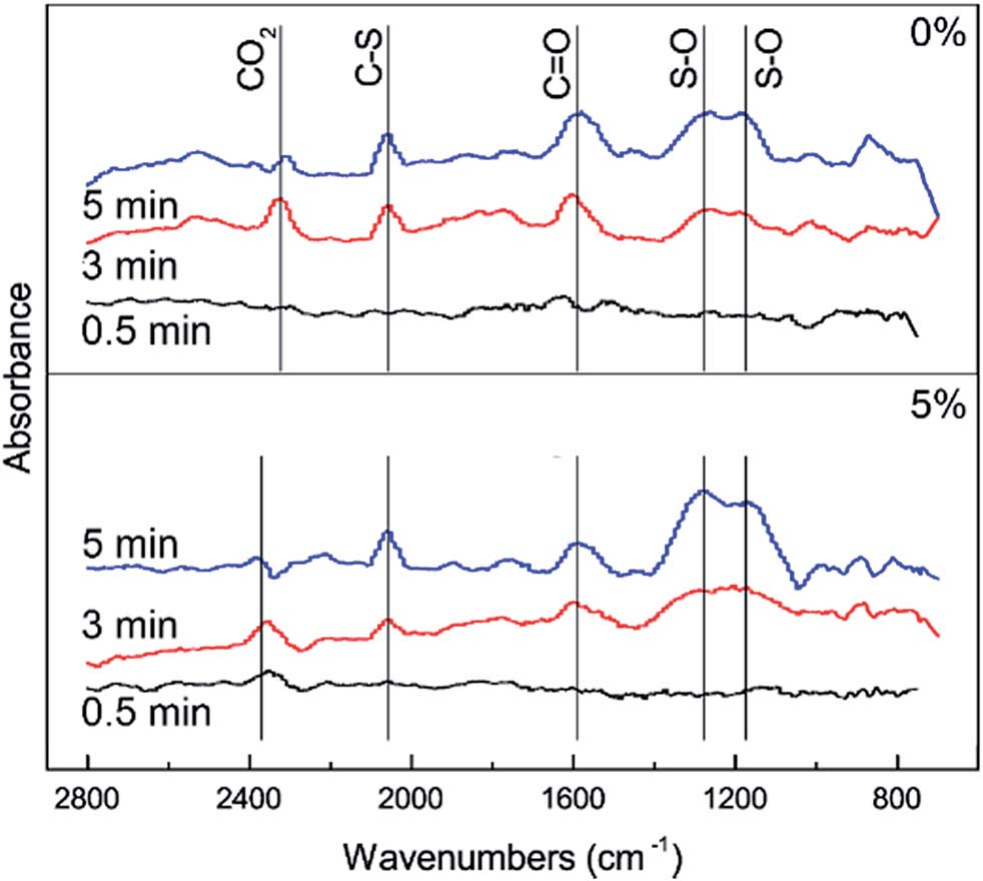
DRIFTS spectra of reactions at different amounts of O_2_ (reaction conditions: 500 ppm H_2_S; 400 ppm COS; 60 ppm CS_2_; reaction temperature = 60 °C; GHSV = 10 000 h^−1^; RH = 49%).

In order to further investigate the effect of different amounts of O_2_ on the conversion of H_2_S, COS and CS_2_ from Cu–Fe/TSAC, DRIFTS measurements of the catalytic reaction over Cu–Fe/TSAC were taken. As shown in Fig. 7, more S–O groups (related to C=O groups) appeared when the reaction was performed below 5% O_2_. This indicated that O_2_ was conducive to the removal of H_2_S due to the oxidation of H_2_S.^27^ Meanwhile, fewer C=O groups appeared when the O_2_ content was 5%, which indicated that O_2_ mainly promoted the oxidation of H_2_S but not the conversion of C=O groups.

### 3.4 Effect of the gas hourly space velocity on the simultaneous removal of H_2_S, COS and CS_2_

**Fig. 8:**
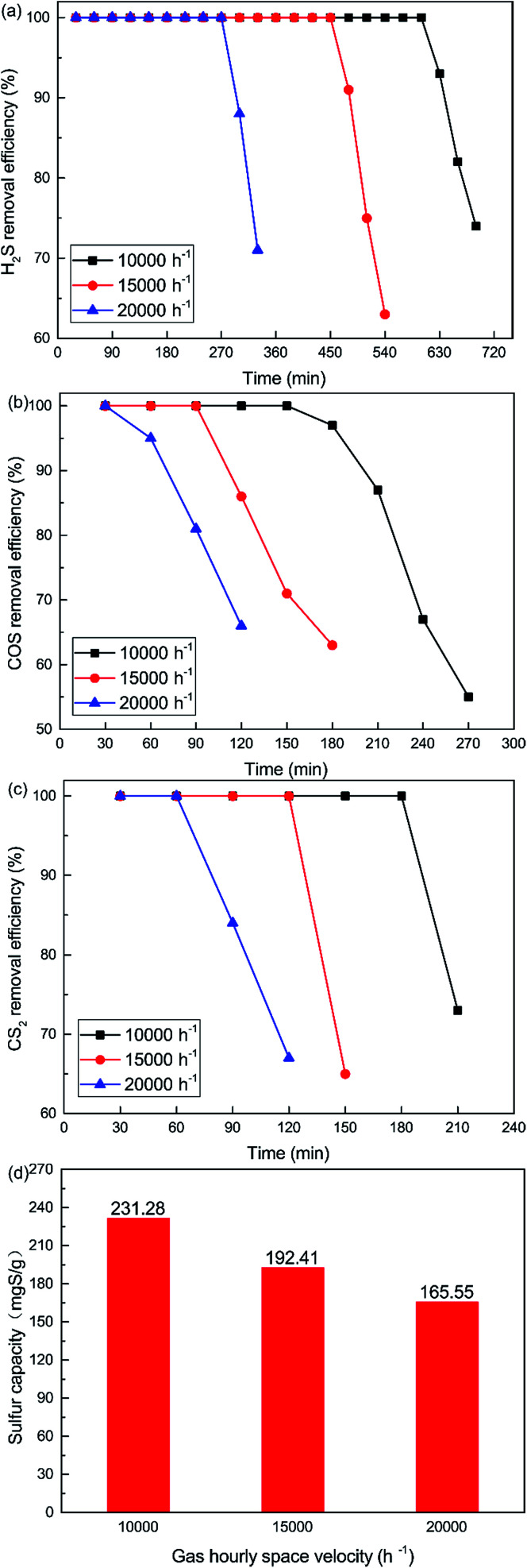
Effect of GHSV on the simultaneous removal of (a) H_2_S, (b) COS, and (c) CS_2_, and (d) sulfur capacity (reaction conditions: 500 ppm H_2_S; 400 ppm COS; 60 ppm CS_2_; reaction temperature = 60 °C; RH = 49%; O_2_ = 0%).

As shown in Fig. 8, the removal efficiency for H_2_S, COS and CS_2_ decreases with increasing GHSV. 100% H_2_S, COS and CS_2_ conversion was maintained for about 600 min, 150 min and 180 min respectively when the GHSV was 10 000 h^−1^. As shown in Fig. 8(d), the sulfur capacity was 231.28 mgS g^−1^ when the GHSV was 10 000 h^−1^. The sulfur capacity decreased with increasing GHSV, and the capacity was 165.55 mgS g^−1^ when the GHSV was 20 000 h^−1^. At low GHSV, more gases could be adsorbed on the surface of the catalyst. As a result, the catalytic hydrolysis and catalytic oxidation reactions could occur fully. However, a high GHSV decreased the contact time between the gases and the catalyst. This led to the decrease of reaction time and conversion efficiency. Meanwhile, a high GHSV was not conducive to the adsorption of gases on the surface of the catalyst. This further decreased the catalytic efficiency.

### 3.5 BET results and reaction process analysis

**Fig. 9:**
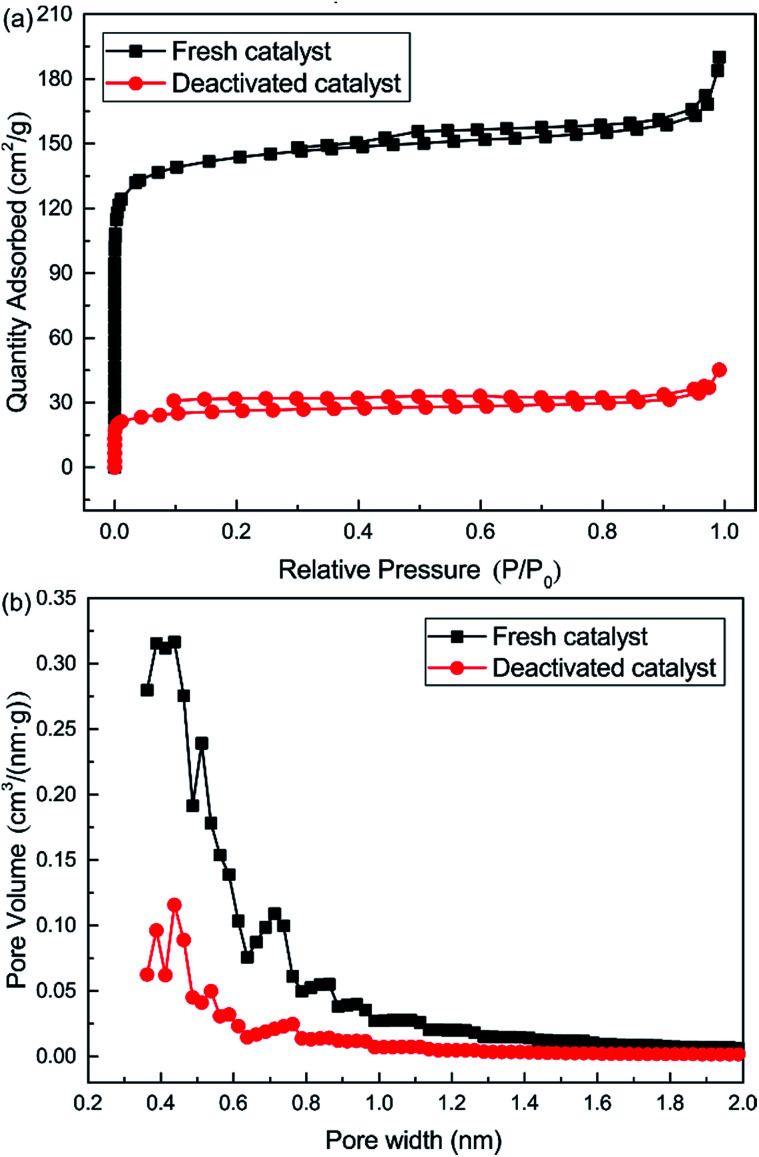
(a) Nitrogen adsorption isotherms and (b) pore size distribution (<2.0 nm) for fresh and deactivated Cu–Fe/TSAC.

**Fig. 10:**
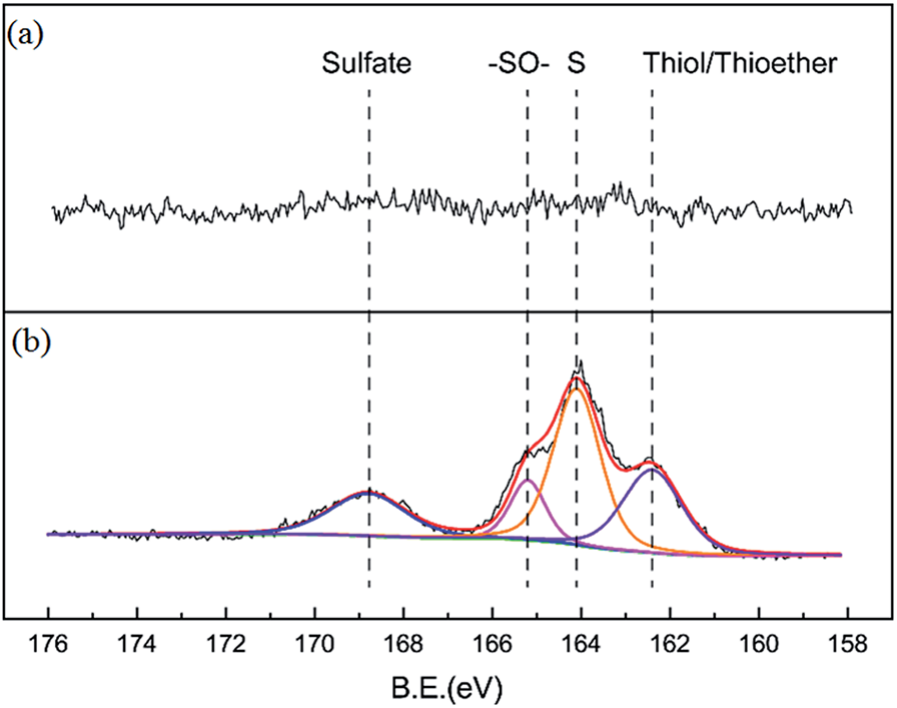
XPS characterization results for (a) fresh and (b) deactivated Cu–Fe/TSAC.

Nitrogen adsorption isotherms and microporous size distribution for fresh and deactivated Cu–Fe/TSAC are shown in Fig. 9 and Table 1. As shown in Fig. 9 and Table 1, Cu–Fe/TSAC had the characteristics of surface area (554 m^2^ g^−1^), microporous volume (0.21 cm^3^ g^−1^) and total pore volume (0.29 cm^3^ g^−1^). The N_2_ adsorption quantity, microporous volume and surface area in the deactivated catalyst obviously decreased, which indicated that the adsorptive ability of the catalyst decreased over time. This affected the desulfurization ability of the catalyst. The XPS characterization results and the data of fresh and deactivated Cu–Fe/TSAC (S2p) are shown in Fig. 10 and Table 2. From the XPS results, it could be found that the thiol/thioether, S, –SO–, and sulfate amounts increased from 0% to 1.92%, 2.90%, 0.83% and 1.25% respectively. This indicated that the deactivation of the catalyst was attributed to the formation of S containing components, such as thiol/thioether, S, –SO– and sulfate. Combined with the BET results, the formation of Cu_2_O led to the decrease of the microporous volume and surface area.

**Table 1:** Porosity parameters for fresh and deactivated Cu–Fe/TSAC

Samples	Surface area (m^2^ g^−1^)	Total pore volume (cm^3^ g^−1^)	Micropore volume (cm^3^ g^−1^)
Fresh catalyst	554	0.29	0.21
Deactivated catalyst	98	0.07	0.04

**Table 2:** XPS data for fresh and deactivated Cu–Fe/TSAC

Sample	Elements	Binding energy (eV)	Amount (%)	Chemical speciation
Fresh catalyst	S2p	—	—	—
Deactivated catalyst	S2p	162.41	1.92	Thiol/thioether
	S2p	164.09	2.90	S
	S2p	165.22	0.83	–SO–
	S2p	168.83	1.25	Sulfate

**Fig. 11:**
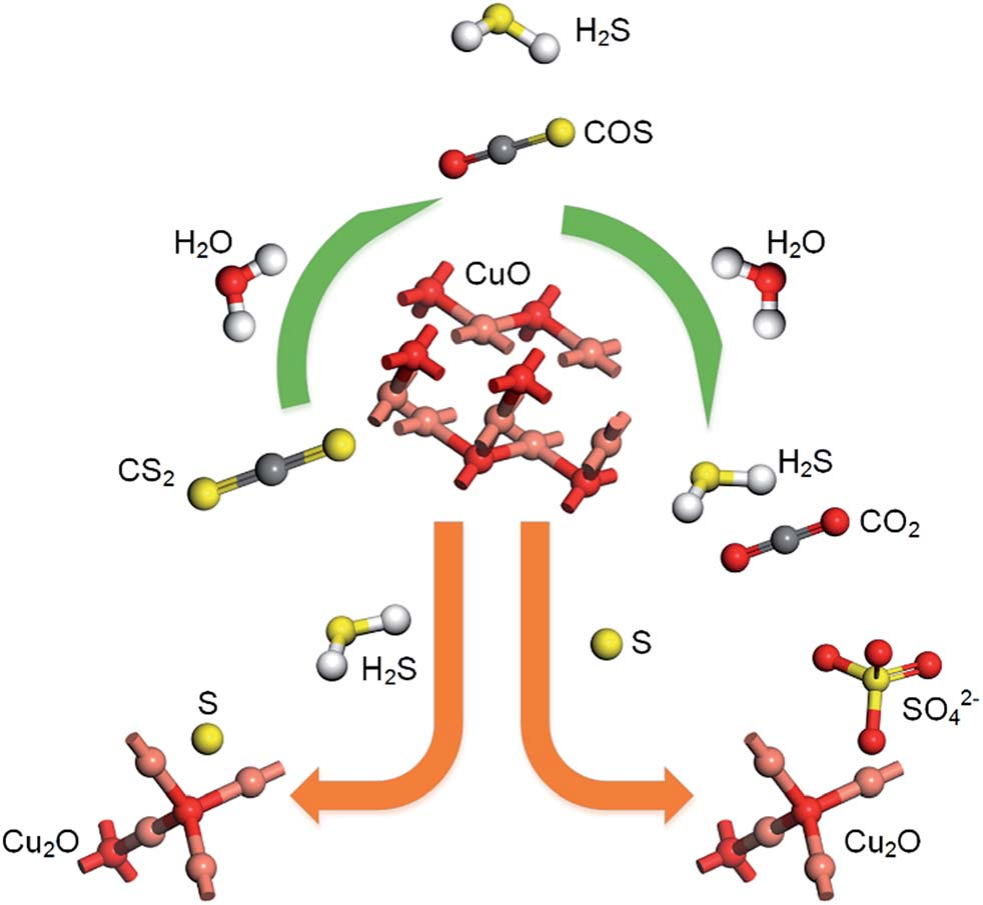
Reaction process diagram.

According to previous experimental results, the removal processes of COS, CS_2_ and H_2_S could be divided into two parts: the catalytic hydrolysis reaction and the catalytic oxidation reaction. In the removal processes, COS and CS_2_ were first hydrolyzed into H_2_S, and then H_2_S was oxidized into S/sulfates. Combined with previous DRIFTS results, the removal processes of COS and CS_2_ were different. For the removal of CS_2_, CS_2_ and H_2_O were first adsorbed on the surface of the catalyst, and then the hydrolysis reaction occurred under the effect of surface functional groups and CuO. However, COS had not been found on the deactivated catalyst, which indicated that gaseous COS directly reacted with adsorbed H_2_O under the effect of surface functional groups and CuO. Furthermore, the conversion process for H_2_S could be regarded as H_2_S → S → SO_2_ → SO_4_^2−^. As mentioned above, the possible reaction process diagram is shown in Fig. 11. As shown in Fig. 11, the main reactions on the surface of the catalyst were as follows.2
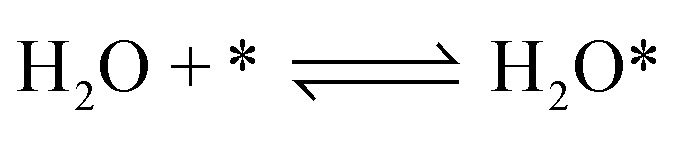
3
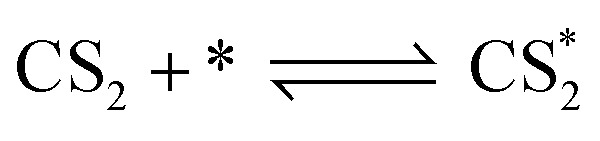
4
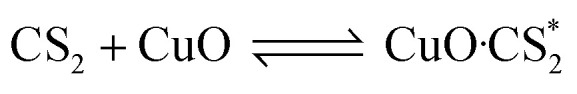
5
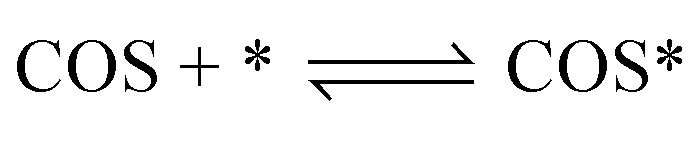
6
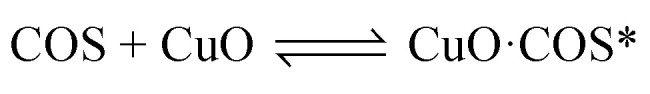
7
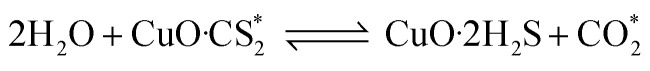
8
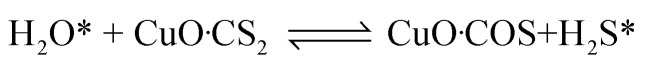
9
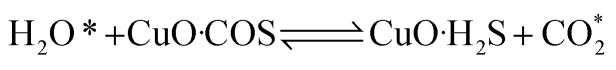
10
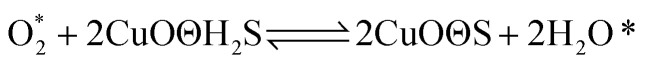
11
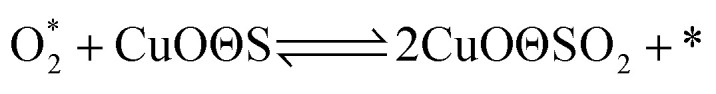
12
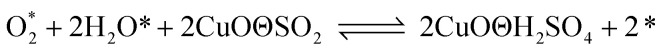


In these reactions, * represents the hydrolysis activity center and Θ represents the oxidation activity center. Eqn (2)–(9) are the catalytic hydrolysis processes and eqn (10)–(12) are the catalytic oxidation processes.

## 4. Conclusions

Modified tobacco stem active carbon (Cu–Fe/TSAC) was prepared by a sol–gel method, and tested for the simultaneous removal efficiency of H_2_S, COS and CS_2_. The influences of reaction conditions for the removal of H_2_S, COS and CS_2_ were investigated. A high reaction temperature improved the hydrolysis and oxidation efficiency, but accelerated the deactivation of the catalyst. DRIFTS results indicated that the deactivation was attributed to the generation of S and sulfates. Excessive water films decreased the diffusion of H_2_S, COS and CS_2_ on the hydrolysis center, and inhibited the catalytic hydrolysis reaction. DRIFTS results indicated that H_2_O promoted the generation of sulfate. Appropriate O_2_ content directly promoted the oxidation of H_2_S, and indirectly promoted the hydrolysis of COS and CS_2_. DRIFTS results indicated that the enhancement of hydrolysis of COS/CS_2_ was attributable to the promotion effect of O_2_ for H_2_S oxidation. A high GHSV decreased the contact time between the gases and the catalyst. Meanwhile, a high GHSV was not conducive to the adsorption of gases on the surface of the catalyst. XPS results indicated that the deactivation of the catalyst was attributed to the formation of S containing components, such as thiol/thioether, S, –SO–, and sulfate. BET results indicated that the adsorptive ability of the catalyst was related to the microporous volume and surface area.

## Conflicts of interest

There are no conflicts to declare.

## Supplementary Material
